# Inside the April 2025 10 Year Anniversary Issue

**DOI:** 10.24908/pocusj.v10i01.19279

**Published:** 2025-04-15

**Keywords:** April 2025, commentary

Dear Readers,

POCUS Journal was founded in 2015 by Dr. Amer Johri and his colleagues at Queen's University in Kingston, Ontario, Canada. Under the umbrella of the Cardiovascular Imaging Network at Queen's (CINQ) and Cinquill, POCUS Journal was first published in 2016. This current issue, published in April 2025, marks the 10th year that the world's leading point-of-care ultrasound journal has been bringing the POCUS community together.

**Figure:**
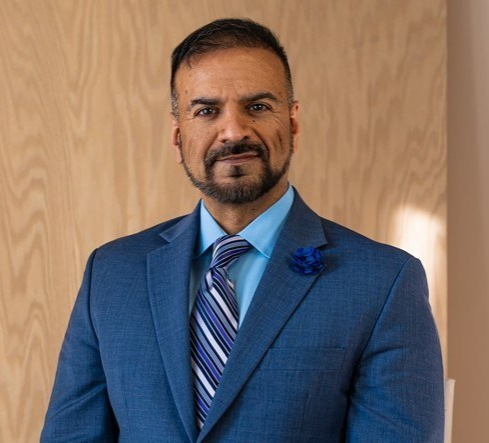
Amer M. Johri, Publisher, POCUS Journal

**Figure:**
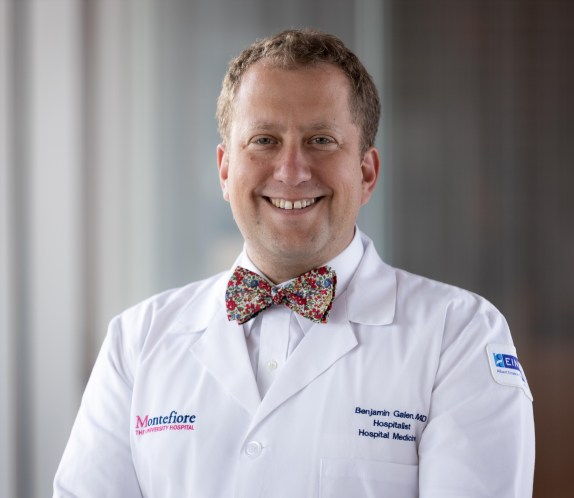
Benjamin T. Galen, Editor in Chief, POCUS Journal

**Figure:**
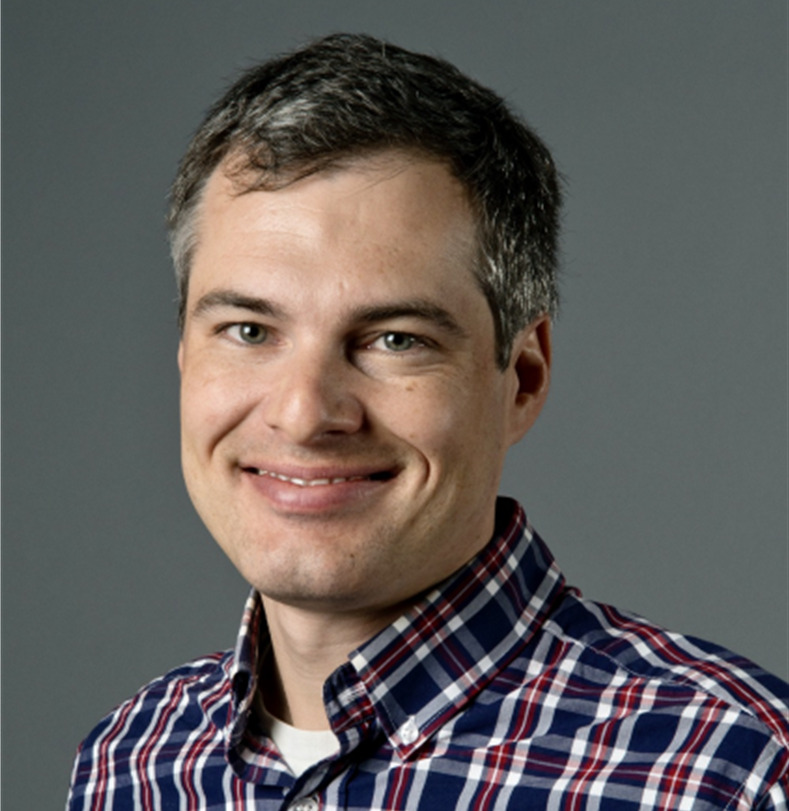
Casey Glass, Deputy Editor, POCUS Journal

You'll notice that the April issue of POCUS Journal features a new cover design as well as a new organization for articles based on organ system. With the growth of our global audience—some articles now reaching over 60,000 users and continuing to grow—we have more confidence, and that is reflected in our new cover with the simple term “POCUS.” We are still the POCUS Journal, but we have successfully embraced this identity.

There are so many high-quality cases, research studies, curricula, and protocols in the April 2025 issue that it is impossible to highlight any single contribution. Together, these manuscripts represent top scholarship by hard working POCUS users from around the world. The POCUS community is very proud that point of care ultrasound enables us to take better, faster care of our patients and we at POCUS Journal are honored to serve this mission.

We are grateful to have such a hardworking and talented editorial board (page 7), whose commitment to the POCUS Journal made this issue possible. We also owe a debt of gratitude to the countless number of peer reviewers who donated their valuable time and insights to improve the articles published herein. We hope you enjoy this issue as much as we do.

Sincerely,

Benjamin T. Galen, MD; Amer M. Johri, MD, MSc, FRCPC, FASE; Casey Glass, MD

